# Post-treatment paradoxical reaction in tuberculous flexor tenosynovitis with rice bodies after a cat bite

**DOI:** 10.1590/S1678-9946202668042

**Published:** 2026-07-03

**Authors:** Zehra Çağla Karakoç, Sait Ada, Önder Pekel, Arzu Avcı, Kubilay Erol, Mahir Kapmaz, Ali Mert

**Affiliations:** 1 Istinye University, Faculty of Medicine, Infectious Diseases and Clinical Microbiology, Istanbul, Türkiye; 2 EMOT Hospital, Division of Hand Surgery, Department of Orthopedics and Traumatology, İzmir, Türkiye; 3 Nişantaşı Pathology Laboratory, Department of Pathology, Istanbul, Türkiye; 4 Tınaztepe University, Faculty of Medicine, Department of Pathology, İzmir, Türkiye; 5 Istanbul Medipol University, Faculty of Medicine, Department of Infectious Diseases and Clinical Microbiology, Istanbul, Türkiye; 6 Istanbul Medipol University, Faculty of Medicine, Department of Internal Medicine, Istanbul, Türkiye

**Keywords:** Paradoxical reaction, Rice bodies, Tenosynovitis, Extrapulmonary tuberculosis, Osteoarticular tuberculosis

## Abstract

Tuberculous tenosynovitis is a rare form of extrapulmonary tuberculosis characterized by an indolent course and nonspecific clinical findings, frequently leading to delayed diagnosis. Paradoxical inflammatory reactions during or after anti-tuberculosis therapy may further complicate clinical management and mimic disease relapse. This is a case study regarding a 54-year-old female veterinarian presented with a four-year history of progressive swelling and induration of the right index finger extending to the palm, accompanied by gradually worsening limitation of finger flexion and hand grasp. Magnetic resonance imaging demonstrated extensive flexor tenosynovitis with synovial proliferation. Tenosynovectomy revealed multiple rice bodies surrounding the flexor tendon. Histopathology showed well-formed granulomas without central necrosis, accompanied by focal stromal coagulative necrosis, while microbiological tests were negative. Tuberculous tenosynovitis was diagnosed based on clinical, radiological, and histopathological findings, and anti-tuberculosis therapy was initiated. The patient showed gradual clinical improvement over the nine-month course of treatment. However, one month after therapy completion, recurrent finger swelling with axillary and new epitrochlear lymphadenopathy developed. In the absence of evidence of relapse, a post-treatment paradoxical inflammatory reaction was suspected. Corticosteroid therapy resulted in rapid clinical improvement. This case highlights the diagnostic challenges of tuberculous tenosynovitis, particularly in patients with delayed presentation and negative microbiological findings, and underscores that paradoxical inflammatory reactions may occur even after completion of anti-tuberculosis therapy, potentially mimicking disease relapse.

## INTRODUCTION

Tuberculosis (TB) remains a major global public health problem. In 2023, about 8.2 million individuals with new or relapsed TB were reported worldwide, with extrapulmonary disease accounting for about 16% of cases^1^. Osteoarticular TB represents 10%–20% of extrapulmonary cases but only 1%–3% of all TB cases. Tuberculous tenosynovitis is a rare manifestation that most commonly affects the flexor tendon sheaths of the hand and wrist and typically follows an indolent course, frequently leading to diagnostic delay. Patients usually present with slowly progressive swelling and limitation of movement, often without systemic symptoms or marked inflammatory markers. Because of these nonspecific findings, the condition may mimic several inflammatory or infectious disorders, and diagnosis is frequently delayed^2,3^.

Rice body formation is an uncommon feature of chronic synovial inflammation and, although initially described in tuberculous arthritis, may also occur in other inflammatory conditions such as rheumatoid arthritis^4^. Magnetic resonance imaging (MRI) may support the diagnosis, but histopathological evaluation remains essential, particularly in culture-negative cases^5,6^.

Paradoxical inflammatory reactions during or after anti-tuberculous therapy represent an important diagnostic challenge. They involve clinical or radiological worsening or new lesions despite appropriate treatment and initial improvement and are thought to reflect immune responses to residual mycobacterial antigens rather than relapse. Although most commonly reported in lymph node and central nervous system TB, data on osteoarticular and musculoskeletal involvement remain limited^7^.

This study reports a case of tuberculous tenosynovitis following occupational exposure to a stray cat bite. The case is notable for delayed diagnosis, multiple rice bodies, and negative microbiological findings. It also illustrates the challenge of a late paradoxical inflammatory reaction occurring about one month after therapy completion.

### Ethics

Written informed consent was obtained from the patient for publication of this case and accompanying images. Ethical approval was not required for this study in accordance with local regulations and institutional policies.

## CASE REPORT

A 54-year-old female veterinarian presented in July 2024 in a hospital in Istanbul, Türkiye, with a four-year history of progressive swelling and induration of the right second finger, extending to the palm, accompanied by gradually worsening limitation of flexion and hand grasp over time, particularly while restraining animals. Pain was relatively mild and was not her main complaint. Her past medical history was notable only for hypertension. She also reported no personal or family history of tuberculosis and no known prior exposure to tuberculosis.

The symptoms began in 2020 after the patient sustained a bite to the right index finger of her dominant hand from a stray cat during occupational exposure while handling the animal without protective gloves. The patient reported that the cat later died despite treatment for a suspected pulmonary disease. Initially, erythema, edema, and local warmth developed at the bite site. A short course of oral amoxicillin–clavulanic acid resulted in partial but incomplete symptom resolution. Within one month, progressive induration extended proximally from the index finger toward the palm, suggesting spread along the flexor tendon sheath. The patient also experienced marked pruritus at the bite site during the early stage, which gradually subsided.

Over the following years, swelling and induration progressively worsened, leading to marked limitation of finger movement and significant functional impairment by the time of presentation, approximately four years after the initial injury.

During this period, several empirical antibiotic regimens were administered, but no sustained clinical improvement was observed. Symptoms gradually progressed, resulting in marked limitation of flexion and extension of the right index finger and hand. No clinical features suggestive of carpal tunnel syndrome were observed.

Diffuse swelling of the right index finger extended from the palm to the distal fingertip were observed during physical examination. Local edema, tenderness, and warmth were present along the flexor tendon sheath. Both active and passive movements were markedly painful and restricted, and the patient could not fully flex the finger (palm-to-fingertip distance ≈6 cm). Right axillary lymphadenopathy (∼1.5 cm) was noted. A Bacillus Calmette–Guérin vaccination scar was present on the left deltoid. The remainder of the examination was unremarkable. Laboratory tests—including leukocyte count, erythrocyte sedimentation rate, C-reactive protein, and liver and kidney function tests—remained within normal limits throughout the four-year course. Chest radiography was normal. Interferon-gamma release assay (T-SPOT.TB) was positive, while HIV serology was negative. Serologic tests for brucellosis (Wright test) and *Bartonella henselae* antibodies were negative.

Magnetic resonance imaging demonstrated extensive multiloculated flexor tenosynovitis of the right index finger extending toward the carpal tunnel, with marked synovial proliferation and relatively low fluid volume, suggestive of chronic granulomatous tenosynovitis (Figures 1A and 1B). Tenosynovectomy was performed due to progressive functional impairment. Marked synovial hypertrophy and numerous rice bodies surrounding the flexor tendon were observed intraoperatively (Figures 2A to 2C).

**Figure 1:**
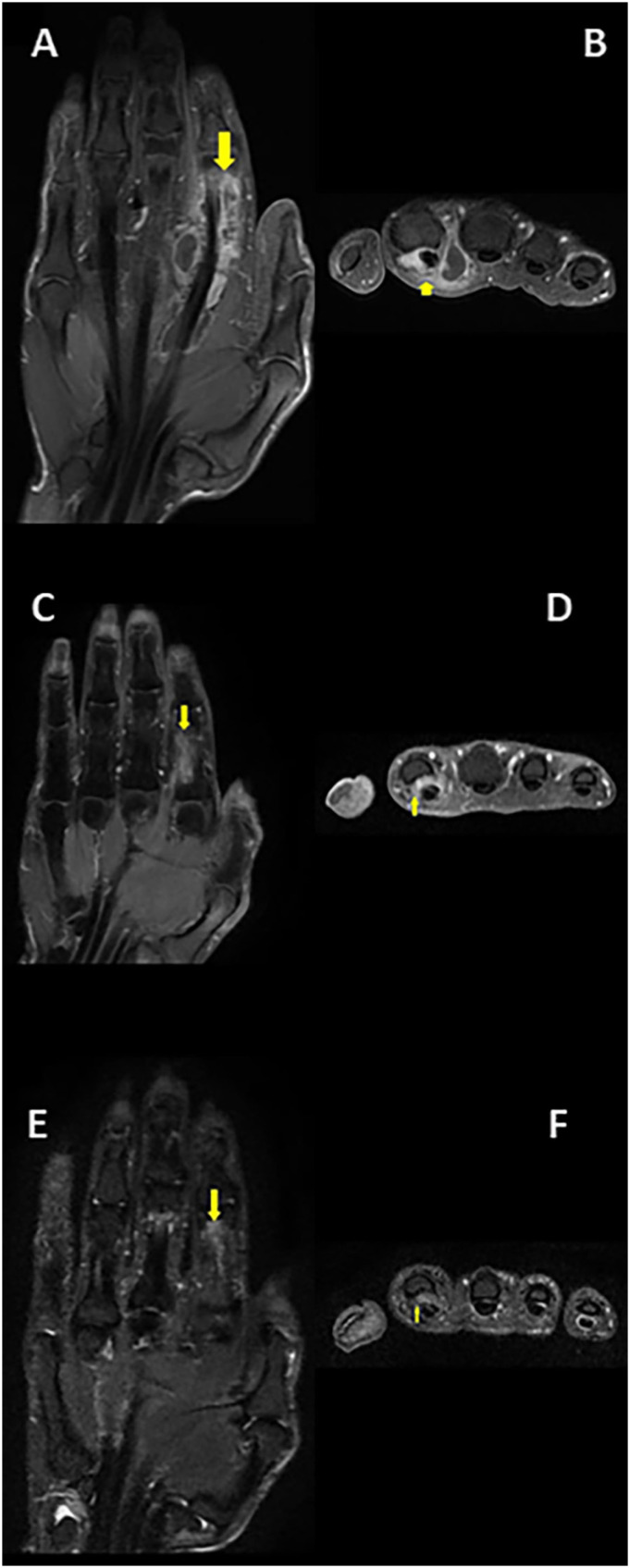
Serial contrast-enhanced fat-suppressed T1-weighted magnetic resonance imaging (MRI) findings in tuberculous flexor tenosynovitis of the second digit of the right hand: (A–F) Coronal and axial images obtained at presentation demonstrate multiloculated synovial thickening surrounding the flexor tendon, extending proximally from the mid-zone at the level of the second phalanx toward the adjacent carpal bones, compatible with loculated tenosynovitis; (C–D) Coronal and axial images obtained after six months of therapy demonstrate residual synovial enhancement between the flexor tendon and adjacent bone at the level of the mid-zone of the second proximal phalanx. Focal cortical erosion of the adjacent bone is also noted; (E–F) Coronal and axial images obtained after nine months of treatment demonstrate further regression of the previously noted synovial hypertrophy adjacent to the second proximal phalanx.

**Figure 2:**
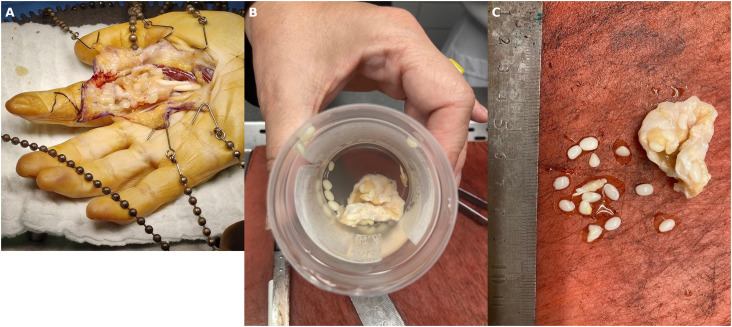
Intraoperative and macroscopic findings in tuberculous flexor tenosynovitis of the second digit of the right hand: (A) Intraoperative view showing extensive synovial hypertrophy and multiple rice bodies surrounding the flexor tendon; (B) Excised synovial tissue with numerous rice bodies; (C) Macroscopic appearance of the removed rice bodies.

Histopathology showed well-formed, non-necrotizing granulomas composed of epithelioid histiocytes and multinucleated giant cells, with focal stromal necrosis; Ziehl–Neelsen, PAS, and Grocott stains were negative (Figures 3A to 3D). Microbiological tests, including acid-fast bacilli smear, mycobacterial culture, real-time PCR for *Mycobacterium tuberculosis* complex, and routine bacterial cultures, were negative. Considering the indolent course, compatible MRI findings (Figures 1A and 1B), and granulomatous histopathology (Figures 3A to 3D), tuberculous tenosynovitis was diagnosed and standard anti-tuberculosis therapy was initiated. After two months, ethambutol and pyrazinamide were discontinued and treatment continued with isoniazid and rifampicin. Follow-up imaging showed progressive regression (Figures 1C and 1D), and therapy was extended to nine months with marked radiological improvement (Figures 1E and 1F).

**Figure 3:**
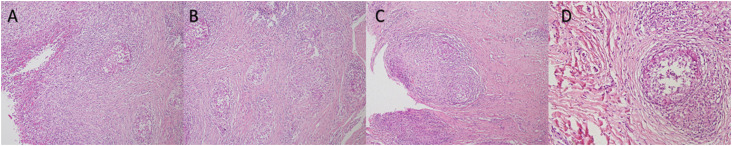
Histopathological findings of the lesion. Histopathological examination revealed isolated, well-formed, non-confluent granulomas without central necrosis. Focal coagulative necrosis confined to the surrounding stromal area is observed in (A). All specimens were stained with hematoxylin and eosin (H&E) and evaluated at magnifications of ×40 (A, B), ×100 (C), and ×200 (D).

One month after completing anti-tuberculosis therapy, the patient developed recurrent swelling, pain, and restricted movement of the right index finger with new epitrochlear lymphadenopathy (∼1 cm) and recurrent axillary lymphadenopathy measuring up to 2 cm. Laboratory findings remained normal and no systemic signs of infection were present. A post-treatment paradoxical inflammatory reaction was suspected. Oral prednisolone was initiated at 32 mg/day (approximately 0.7 mg/kg/day), resulting in rapid clinical improvement. The initial dose was maintained for three weeks, followed by a gradual taper of 8 mg per week; in the final week, 4 mg/day was administered before discontinuation. The total duration of corticosteroid therapy was about two months. No relapse was observed during six months of follow-up after discontinuation of corticosteroid therapy.

## DISCUSSION

Tuberculous tenosynovitis is a rare form of extrapulmonary tuberculosis and represents a small proportion of musculoskeletal tuberculosis cases. The disease typically follows an indolent course and frequently leads to diagnostic delay^2^,^3^. In our patient, symptoms persisted for nearly four years before a definitive diagnosis was established, consistent with previous reports describing prolonged disease duration.

Zoonotic transmission of *Mycobacterium bovis* from animals to humans, including genetically confirmed cat-to-human transmission, has been reported^8^,^9^. In this case, a stray cat bite preceding the onset of chronic localized disease raised suspicion of possible zoonotic exposure. The animal reportedly developed respiratory illness and died shortly after the bite, suggesting a potential mycobacterial infection. However, this possibility was not initially considered, and specific culture conditions for *M. bovis* were not requested. This also reflects real-life clinical practice, in which zoonotic tuberculosis is rarely suspected at initial evaluation. Since *M. bovis* growth may be inhibited on conventional glycerol-containing Löwenstein–Jensen media, which may reduce microbiological yield, confirmation could not be obtained. This represents an important diagnostic limitation in our case. The use of such media may have led to false-negative culture results. Therefore, the lack of microbiological confirmation does not exclude zoonotic tuberculosis. This underscores the importance of selecting appropriate culture conditions in patients with compatible exposure.

Non-tuberculous mycobacterial infections, particularly *Mycobacterium marinum*, should be considered in patients with chronic tenosynovitis and negative microbiological findings. However, in our patient, the indolent clinical course, granulomatous histopathology, and sustained response to anti-tuberculosis therapy supported a presumptive diagnosis of tuberculous infection. Although *M. marinum* remains an important differential diagnosis, particularly given its indolent course and predilection for the upper extremities, the absence of a clear history of aquatic exposure and the presence of a preceding cat bite make this etiology less likely in our case. However, environmental exposures are not always recognized or recalled, and diagnosis can be challenging due to the need for specific culture conditions, including lower incubation temperatures, as well as prolonged incubation times and limited microbiological yield, which may limit definitive exclusion^10^.

Similar cases of chronic tenosynovitis involving both non-tuberculous and tuberculous mycobacteria have been increasingly reported in the literature. In particular, infections caused by *Mycobacterium avium* complex and *Mycobacterium abscessus*, as well as tuberculous tenosynovitis, may have overlapping clinical features such as indolent progression, negative microbiological findings, and delayed diagnosis. These observations highlight the diagnostic complexity of chronic tenosynovitis and the importance of maintaining a high index of suspicion in atypical or culture-negative cases^11-13^. Other infections associated with cat exposure, including *Pasteurella multocida* and *B. henselae*, were considered unlikely based on negative serology and lack of response to empirical antibiotic therapy^14^.

Imaging also played an important role in both diagnosis and follow-up. MRI enabled detailed assessment of disease extent and proved useful for monitoring treatment response. Typical MRI findings include synovial thickening and proliferation with relatively limited fluid and heterogeneous T2 signal intensity^15^. In our patient, serial MRI examinations helped guide clinical management despite the absence of microbiological confirmation.

Rice body formation is a characteristic but nonspecific feature of chronic synovial inflammation and is most commonly associated with tuberculosis and rheumatoid arthritis. These structures are thought to result from synovial microinfarction followed by fibrin deposition and are typically observed in long-standing disease. In our patient, the prolonged clinical course and extensive synovial involvement were consistent with this mechanism. Notably, rice bodies were not clearly visualized on MRI and were identified only during surgery. This highlights an important limitation of imaging, as small or clustered rice bodies may be difficult to detect; therefore, their absence on MRI does not exclude the diagnosis^4^.

Histopathological examination revealed well-formed granulomas composed of epithelioid histiocytes and multinucleated giant cells with focal stromal coagulative necrosis. When interpreted together with compatible clinical and radiological findings, these features support the diagnosis of tuberculosis. Classical caseous necrosis may be absent in extrapulmonary tuberculosis, particularly in small biopsy specimens^5^,^6^.

In our patient, microbiological investigations were negative; however, this does not exclude tuberculosis, as musculoskeletal disease is typically paucibacillary and microbiological confirmation is often difficult^6^,^16^. The indolent clinical course, compatible MRI findings, granulomatous histopathology, and the favorable response to anti-tuberculosis therapy collectively supported the diagnosis of tuberculous tenosynovitis.

Treatment of musculoskeletal and tendon sheath tuberculosis often requires prolonged therapy because of the relatively low vascularity of affected tissues and limited drug penetration. For this reason, treatment duration is frequently individualized, particularly in patients with delayed diagnosis or extensive synovial involvement, which may increase the risk of relapse. Some authors have suggested extending therapy up to 18 months in selected cases^3^,^17^. Our patient had symptoms for nearly four years before diagnosis, and extensive tenosynovial involvement was observed. Therapy was therefore extended to nine months and was considered adequate based on the progressive clinical and radiological improvement.

Paradoxical inflammatory reactions are defined as clinical or radiological worsening after initiation of anti-tuberculosis therapy despite appropriate treatment and microbiological control. Most reactions occur within the first one to three months of therapy and are thought to reflect an exaggerated immune response to mycobacterial antigens. Although initially described in HIV-associated tuberculosis, paradoxical reactions are increasingly recognized in HIV-uninfected patients and appear more frequent in extrapulmonary disease than in pulmonary tuberculosis (approximately 25% vs. 2%, respectively). Lymph nodes are the most commonly involved site, followed by central nervous system involvement. Paradoxical reactions occurring after completion of therapy, however, are uncommon^7^,^18-20^.

Data on post-treatment paradoxical reactions in tuberculous tenosynovitis are very limited. In our patient, axillary lymphadenopathy that had resolved during therapy recurred shortly after treatment completion, together with new epitrochlear lymphadenopathy. In the absence of clinical evidence of relapse, these findings were interpreted as a paradoxical inflammatory reaction. The rapid response to adjunctive corticosteroid therapy further supported this interpretation. Corticosteroids may be considered in selected cases to control inflammation and prevent functional impairment, although careful differentiation from treatment failure or drug resistance remains essential^7^.

Despite the clinical findings suggesting a post-treatment paradoxical reaction, the absence of histopathological confirmation is an important limitation, and the diagnosis remains presumptive. Alternative explanations, including relapse, superimposed infection, or other inflammatory conditions, cannot be fully excluded. A repeat biopsy was not performed due to the lack of systemic signs of infection, normal laboratory findings, and the patient's rapid response to corticosteroid therapy. As noted in previous studies, diagnosis in such cases often relies on clinical and radiological findings together with treatment response; however, this approach limits diagnostic certainty^7^.

## CONCLUSION

This case highlights several important clinical points. Tuberculosis should remain in the differential diagnosis of chronic tenosynovitis of the hand even in the absence of systemic symptoms or microbiological confirmation. A detailed exposure history, including occupational and animal contact, may provide valuable diagnostic clues. The presence of rice bodies can support the suspicion of tuberculous tenosynovitis. Finally, paradoxical inflammatory reactions should be recognized as a potential cause of clinical worsening after treatment, and corticosteroid therapy may be beneficial in selected patients.

## Data Availability

The complete anonymized dataset supporting the findings of this study is included within the article itself.
